# Petri Net-Based Model of *Helicobacter pylori* Mediated Disruption of Tight Junction Proteins in Stomach Lining during Gastric Carcinoma

**DOI:** 10.3389/fmicb.2017.01682

**Published:** 2017-09-06

**Authors:** Anam Naz, Ayesha Obaid, Faryal M. Awan, Aqsa Ikram, Jamil Ahmad, Amjad Ali

**Affiliations:** ^1^Atta-ur-Rahman School of Applied Biosciences, National University of Sciences and Technology Islamabad, Pakistan; ^2^Research Center for Modeling & Simulation, National University of Sciences and Technology Islamabad, Pakistan

**Keywords:** *Helicobacter pylori*, gastric cancer, post translational modifications (PTMs), tight junction (TJ) proteins, phosphorylation sites, petri net (PN) models

## Abstract

Tight junctions help prevent the passage of digestive enzymes and microorganisms through the space between adjacent epithelial cells lining. However, *Helicobacter pylori* encoded virulence factors negatively regulate these tight junctions and contribute to dysfunction of gastric mucosa. Here, we have predicted the regulation of important tight junction proteins, such as Zonula occludens-1, Claudin-2 and Connexin32 in the presence of pathogenic proteins. Molecular events such as post translational modifications and crosstalk between phosphorylation, O-glycosylation, palmitoylation and methylation are explored which may compromise the integrity of these tight junction proteins. Furthermore, the signaling pathways disrupted by dysregulated kinases, proteins and post-translational modifications are reviewed to design an abstracted computational model showing the situation-dependent dynamic behaviors of these biological processes and entities. A qualitative hybrid Petri Net model is therefore constructed showing the altered host pathways in the presence of virulence factor cytotoxin-associated gene A, leading to the disruption of tight junction proteins. The model is qualitative logic-based, which does not depend on any kinetic parameter and quantitative data and depends on knowledge derived from experiments. The designed model provides insights into the tight junction disruption and disease progression. Model is then verified by the available experimental data, nevertheless formal *in vitro* experimentation is a promising way to ensure its validation. The major findings propose that *H. pylori* activated kinases are responsible to trigger specific post translational modifications within tight junction proteins, at specific sites. These modifications may favor alterations in gastric barrier and provide a route to bacterial invasion into host cells.

## Introduction

Highly organized intercellular tight junctions (TJ) are crucial structural components of the intact epithelium architecture and provide protection against intruding pathogens. Disruption of these epithelial barriers is an important hallmark of *Helicobacter pylori*-dependent inflammation and neoplastic tissue transformation (Wessler and Backert, 2008). *Helicobacter pylori* is known for selectively colonization of the hostile environment such as gastric mucosa. The mucus layer within gastric mucosa remains in close contact with the epithelial cells at the apical side of the intercellular contacts. *H. pylori* actively interferes the host cells and exerts an astounding set of strategies to manipulate these epithelial cell-to-cell junctions. This negative interaction between the pathogen and host results into major consequences such as altered cell polarity, migration and invasive growth as well as pro-inflammatory and proliferative responses (Ashida et al., 2012).

The *H. pylori* through type IV secretion system (T4SS) inject the virulence factor cytotoxin-associated gene A (CagA) (Terradot and Waksman, 2011). However, to take charge of host cell it adapts other techniques as well to intrude the cells; mostly by loosening the TJ in the epithelial lining (Amieva et al., 2003). Normally, post translational modifications (PTMs) can affect the structure and function of these TJ proteins. There are many reports showing the role PTMs of in dysregulation of normal genes and their promotors for initiation and progression of infection and diseases (Parsonnet et al., 1991; Akhtar et al., 2001; Blaser and Berg, 2001; Perri et al., 2007). Pathogens alter the behavior of proteins to change the dynamics as per its desire. Thus, exploiting these facts the detail mechanism of action behind de-/regulation of these changes within host cells could be determined by integrative approaches.

The significant TJ proteins in epithelial lining include claudins, occludins, connexins, junction-adhesion molecules (JAMs) as well as membrane associated proteins such as zonula occludens (ZO-1, -2, -3) (Alberts et al., 2002). Claudins, connexins and occludins along with their adapter proteins are mostly targeted by the pathogen for dysregulation, therefore, factors regulating the normal functioning of these proteins were explored to elucidate the possible reasons of their disruption. These proteins have been previously reported to play important roles in tight junction barrier deficits induced by *H. pylori* (Amieva et al., 2003; Song et al., 2013; Wang et al., 2014). Claudin-2 (CLDN2), Connexin32 (CX32), and ZO-1 are focused in current study to evaluate for their possible modifications by PTMs and hence the negative regulation of their functions

CLDN2, usually located in gut epithelia, helps in pore formation, thus regulates paracellular transport through epithelial cells (Rosenthal et al., 2010). Its over expression has also been linked to *H. pylori*-induced inflammatory bowel disease, ulcer and carcinoma (Randall et al., 2016). Similarly, a gap junction protein CX32, found in the epithelium of the gastrointestinal tract (GIT), when mislocalized or having altered function could lead to gastric carcinoma (Jee et al., 2011). ZO-1 has already been found at mature tight junctions with altered function and structure causing serious barrier defects specifically by *H. pylori* as explored by Fiorentino et al. (Fasano, 2000; Fiorentino et al., 2013). Similarly, dysregulated claudin-1/-2/-4 are also found to be involved in a number of benign bowel inflammatory disorders characterized by mucosal barrier dysfunction (Wardill et al., 2014). Besides that, pathogen also targets specific kinases, cytokines and enzymes to induce specific PTMs within TJ proteins for their survival and reproduction within the host cells (Maeda et al., 2000; Rad et al., 2004; Amieva and El–Omar, 2008). These modifications also contribute to On and Off the intracellular signaling, among modifications the kinase specific phosphorylation is the most wide spread and well-studied fact (Awan et al., 2014). Increased tyrosine phosphorylation of ZO-1 and decreased expression leading to TJ disruption and allowing the entry of foreign particles to enter the cell (Martin and Jiang, 2009). Similarly, occludin tyrosine phosphorylation has been found to be related with its disassociation with ZO-1 leading to the disturbed junctional complex (Lee, 2015). The dysregulated phosphorylation of many host signaling proteins (MLC, CLDN4, CLDN5) have also been reported to be linked with gastritis and even gastric carcinoma (Martin and Jiang, 2009). Similarly, aberrant methylation of promoters and genes plays a biologically significant role in carcinogenesis. Methylation of some promoters of genes has already been reported in progression of *H. pylori* infection and even gastric carcinoma (Niwa et al., 2010). Methylation is one of the significant modifications that can alter the expression, function and effect of proteins within signaling pathways, which can lead to the disease onset when modulated by pathogens. Thus, the overall importance of these barrier proteins along with potential PTM sites has been realized to maintain the cell integrity, polarity and normal growth. The study also focuses on the prediction of potential kinase targeted sites for phosphorylation within TJ proteins, which can be earmarked by the pathogen to alter junction mechanism for its entry.

The most important CagA mediated infection pathway is modeled through Hybrid Petri Nets (HPNs) to understand the dynamics of infection and disease progression. The changes in the behavior of key entities (such as kinases, cytokines: NF-κB, ILs, TJ proteins, etc.) and difference in their relative levels (expression/concentration) before and after infection have been observed through a step-wise simulation experiments. Our study focuses on developing a qualitative integrated model to decipher the detail mechanism triggered by over-expression of IL1β and IL8 leading to dysregulated kinase specific phosphorylations within TJ proteins. HPNs was adopted because of its high level of integration and recognition as a powerful modeling tools for efficient modeling and analysis of biological pathways (David and Alla, 2010). This formal basis combined with the nice graphical representation makes it possible to argue about processes, and thereby enables the possible establishment of certain patterns. Moreover, they can represent the system behavior even when the biological mechanism is not fully understood, by combining different levels of abstraction in a single model and enable users to verify system properties, verify system soundness, and simulate the dynamic behaviors (Matsuno et al., 2003). To verify and evaluate the effect of *H. pylori* proteins on host cells and pathways, we have modeled both the normal and diseased conditions and to look for difference in the expression of proteins in both cases. The predicted behavior outcomes from the models are in line with experimental findings of others (Table 1), thus predicted the dynamic behavior of proteins without extensive wet lab experiments and computationally expensive parameter estimation.

**Table 1:** Summary of comparison between reported experimental observations and simulation results.

**Observed proteins**	**Experimental observations**	**Model predictions**	**References**
CLDN2	+	+	Aung et al., 2006; Song et al., 2013
CX32	−	−	Jee et al., 2011; Wang et al., 2014, 2015
ZO-1	−	−	Amieva et al., 2003; Ma et al., 2004; Ashida et al., 2012
IL8	+	+	Noach et al., 1994; Nagashima et al., 2015; Ferreira et al., 2016
IL1B	+	+	Noach et al., 1994; Harris et al., 1996
NF-κB	+	+	Keates et al., 1997; Ma et al., 2004
ERK	+	+	Meyer-ter-Vehn et al., 2000; Lee et al., 2010
MAPK	+	+	Churin et al., 2003; Nishioka et al., 2003
P38	+	+	Takahashi et al., 2001; Nakayama et al., 2004

We propose here the mechanism of action behind these alteration of epithelial barrier (TJ proteins) induced by *H. pylori* through specific PTMs. Also, a comprehensive pathway model built effectively illustrated the key regulatory mechanisms of TJs and how they respond to *H. pylori* infection. The integrated structural and mathematical modeling approach applied here helped in establishing the bacterial and host epithelial interaction and the constituents involved in improvising the epigenetic changes via PTMs in TJ proteins which ultimately leads to gastric epithelial cell barrier dysfunction.

## Materials and methods

**Figure 1:**
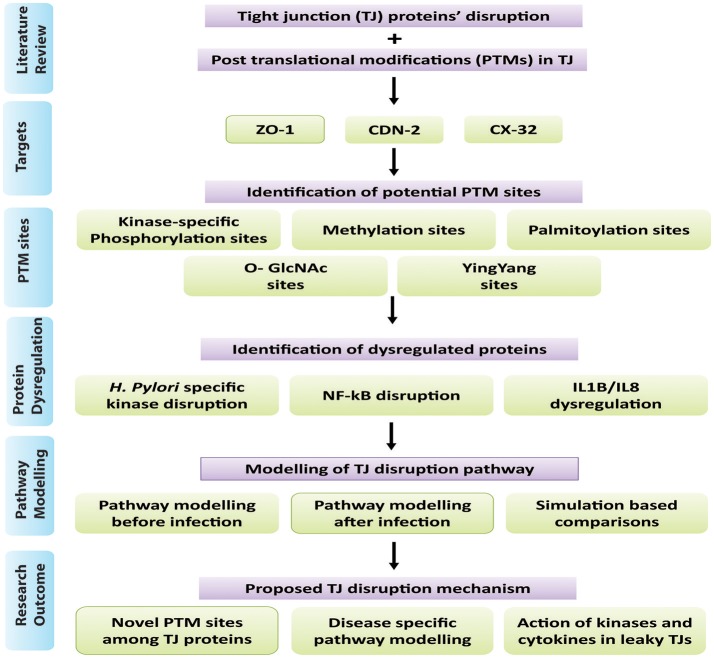
Overview of methodology: Summary of steps followed in this study has been shown in the form of flow diagram. Major steps include literature view, prioritization of target proteins, and identification of PTM sites within selected TJ proteins, identification of dysregulated proteins/kinases after infection and modeling of infection pathway leading to barrier disruption.

The study has been broadly categorized into two main parts: (1) Protein analysis, where all three target proteins (CLDN2, CX32, and ZO-1) were analyzed using their sequences and structures. Proteins were then scrutinized for potential PTM sites regulated by important epigenetic mechanisms, which can be induced by the pathogen during infection. Subsequently, specific kinase proteins were prioritized that can be triggered by the pathogen and their targeted residues. (2) The signaling pathways disrupted by dysregulated kinases and PTMs were studied. Based on these altered pathways, an abstracted mathematical model was designed as a baseline for further *in silico* experimentation. Applying PN approach, a dynamic model was constructed which provides biological insights for *H. pylori* related TJ disruption and dynamic regulation of various signaling proteins during infection. An overview of the approach followed in the current study has been shown in Figure 1 and each step is explained accordingly.

### Proteins' analyses

#### Sequences, structures and conservation analysis of TJ proteins

FASTA sequences of human proteins ZO-1, CLDN2 and CX32 were retrieved from Swiss-Prot database (Boeckmann et al., 2003) with primary accession numbers of Q07157, P57739 and P08034, respectively. To get the homologs of the selected proteins, BLASTp (Altschul et al., 1997) was performed against few organisms with higher bit scores, and *E* ≤ 0 avoiding any synthetic constructs, isoforms and unnamed proteins to get conserved sites among these proteins across species. Homologs of ZO-1 from *Mus musculus* (P39447) and *Canis familiaris* (O97758) are collected. Selected sequences for CLDN2 were from *Canis familiaris* (Q95KM6), *Mus musculus* (O88552), and *Bos taurus* (Q765P1). For CX32, homologs were retrieved from *Rattus norvegicus* (P08033), *Mus musculus* (P28230), *Cavia porcellus* (Q8K4M7) and *Bos taurus* (O18968). Homologs for each protein were then aligned using CLC workbench (Workbench, 2010) to get conserved regions and sites amongst them. In order to predict membrane spanning regions and their orientations within CLDN and CX32 TMHMM Server v. 2.0 (Krogh et al., 2001) and TMPred Server (Hofman, 1993), were employed. The 3D models were constructed to explore regions having potential to form helices embedded within the membrane, as they seal the intracellular space to maintain TJ integrity (Van Itallie and Anderson, 2013).

#### Prediction of sites prone to post-translational alterations

Various modification sites including kinase specific phosphorylation, methylation, palmitoylation and O-GLcNAc were predicted within CLDN2, CX32, and ZO-1. Methylation sites among them were estimated using PMes program which predicts the potential methylation sites by analyzing protein sequence, position of residues and their physicochemical properties with structural characteristics (Shi et al., 2012). This feature increases the robustness and accuracy of this tool as compared to other methylation prediction methods. CSS-PALM 4.0 (Ren et al., 2008), an online tool following a robust clustering and scoring strategy (CSS) algorithm was used to identify palmitoylation sites within candidate proteins. O-GlcNAc and Yin-Yang sites were predicted using YinOYang 1.2 program (Gupta and Brunak, 2002). Phosphorylation sites for each serine (Ser) and threonine (Thr) and tyrosine (Tyr) residues were predicted using Netphos 2.0 (Blom et al., 1999), based on artificial neural network programs, out of which exposed kinase specific sites were retrieved using NetphosK (Blom et al., 2004), KinasePhos 2.0 (Wong et al., 2007), and GPS 2.1 (Xue et al., 2010). Sites verified by two or more databases were selected. Obtained results were then scanned manually for experimentally verified sites within literature and Phospho. ELM database (Diella et al., 2004) was also consulted to identify experimentally validated phosphorylation sites. To prioritize sites exposed (Surface accessibility) for kinases NetSurfP (Petersen et al., 2009) and TMHMM server (Krogh et al., 2001) were employed. Finally, surface exposed kinase specific phosphorylation sites were prioritized which have not been previously reported in case of *H. pylori* induced infection which were then checked for kinases action.

#### Prioritization of modification sites targeted by disease specific kinases

Identifying kinase substrate and their cognate phosphorylation sites is fundamental to reveal the molecular mechanism of disease progression. Thus, to particularize the selected phosphorylation sites in previous steps, we manually listed those kinases that are specifically targeted by *H. pylori* infection exploring published experimental data. Sites targeted by these specific kinases were then prioritized for further analysis. Dysregulation of some specific kinases lead to PTMs resulting in crucial epigenetic changes within TJ proteins and also alter important signaling pathways that ultimately regulate epithelial cell polarity.

### Disease induced signaling pathway analysis

#### Identification of *H. pylori* induced signaling pathways targeting disease specific kinases

**Figure 2:**
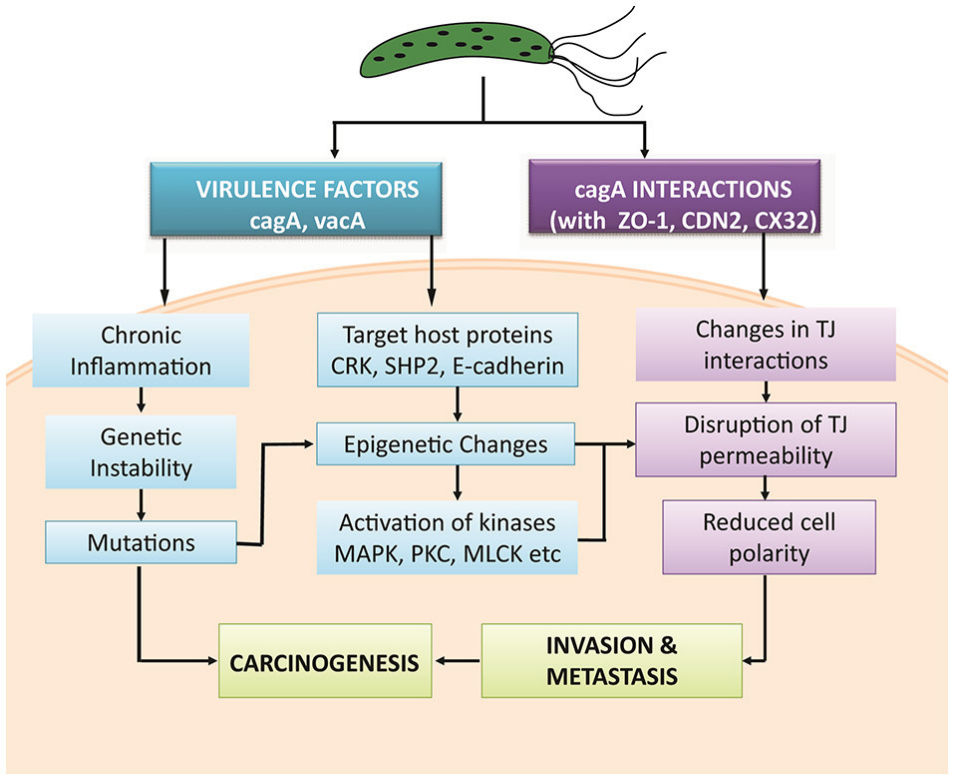
Possible mechanisms adapted by *H. pylori* and its virulence factors to induce gastric carcinoma.

TJs are modulated by intra cellular signaling pathways (Matter and Balda, 2003) which when dysregulated in response to *H. pylori* infection, affects the epithelial barriers. Literature survey was performed to investigate how phosphorylation can influence a series of biological pathways to regulate TJ molecules in human epithelial cells under normal and pathological conditions. Possible mechanisms targeted by pathogen are mapped to predict routes adapted by pathogen leading to gastric carcinoma (Figure 2).

One of the important factor to reveal the regulation of signaling pathways and major protein functions is the identification of kinases, enzymes and their precise targeted phosphorylation sites. Computational predictions include selection of protein sequence, prediction of the phosphorylations sites, cellular context of kinases and signaling pathways affected by them. Based on these considerations, we propose a probabilistic model to predict a pathway induced by *H. pylori* infection to stimulate PTMs within TJ proteins and resultant altered behavior.

#### Mathematical modeling of proposed mechanism adopted by *H. pylori* to induce barrier dysregulations

##### Modeling approach

The current study employs PN approach already explained by Obaid et al. (2015) to study the dynamics of signaling pathways followed by *H. pylori* during infection (Ruths et al., 2008). Hybrid Petri Nets (HPNs) can be defined as a type of PNs that describe the level of activation and inhibition of a particular gene/protein activity and even the dynamics of whole network governing their concentrations. Within HPNs thresholds can be maintained to define the activation and deactivation of entities, thus can demonstrate both continuous and discrete elements. Therefore, it is an efficient modeling approach able to handle all types of biological factors. Thus, in our study, we also demonstrated HPNs to translate biological facts involved in regulation of tight junction proteins by kinases qualitatively, without explicit knowledge of quantitative network dynamics.

A HPN is a directed bipartite graph, which is a 3-tuple (P, T, W), with Places (resources/entities), Transitions (processes), and Weighted arcs (Directed arrows are arcs or edges which connect only places to transitions and vice versa). In a model, circles show places, whereas boxes or bars represent the transitions. Arcs weights usually represent the multiplicity and by default its value remain 1. Model simulates by firing a transition which represents the withdrawal of tokens from the input place and following the arc multiplicities deposits it to the output places (David and Alla, 2010). The steps involved in the HPN model generation are; (1) literature survey to extract the possible route for activation of particular kinases; (2) iterative abstraction of the extracted pathway; (3) construction of model, (4) analysis of the model and verification of the predictions.

##### HPN model generation

In this study, a qualitative HPN model was designed using SNOOPY v 2.0 (Rohr et al., 2010) tool to study the regulation (upstream and downstream) of TJ proteins based on kinase specific actions. Generated simulations actually determine the relationship between continuous and discrete entities. Two models have been generated to compare and validate the behaviors during normal conditions and diseased condition. Places in both models represent receptors, proteins and kinases whereas transitions illustrate the processes (e.g., gene enhancing, biological reactions, de-/activation, complex formation, PTMs, epigenetic changes, etc.). As comprehensive knowledge of kinetic parameters is mostly unavailable for networks, therefore, because of limited applicability, quantitative models are quite complex to model. We here applied Prior knowledge network (PRN) approach to construct the model, based on non-parametric strategy. As the network connectivity is sole determinant of signal flow through the system, our model relies on relative concentrations of the proteins (up-/down-regulation) and not the absolute values. As the model is qualitative and abstracted in nature, it potentially limits the complex transcriptomics data, only indicating the occurrence of interactions between proteins.

##### Model verification

Biologically, a reaction can occur if its reactants and conditions fit certain criteria, similarly, in PN models a transition can be fired to get activated with a certain transition speed based on defined parameters (tokens, arch weights, inhibition, etc.). The designed models are therefore verified for both normal and disease conditions by comparing the obtained simulation results with the already available expression data of protein and kinases activation. In brief, the activity levels of each entity within our models and changes in their level over time correlate with the concentration of active entities within a cell. Correlation of designed models with experimental studies verified the reliability of our model and hence it can further provide biological insights related to *H. pylori* infection, disruption of TJ proteins and entry of pathogen within host cell. Following this methodology various other aspects of disease prevalence, host-pathogen interactions, factors aiding pathogen survival and hijacking of immune system can also be explored prior to expensive and time taking experimental methods.

#### Abstraction of the altered signaling pathways

**Figure 3:**
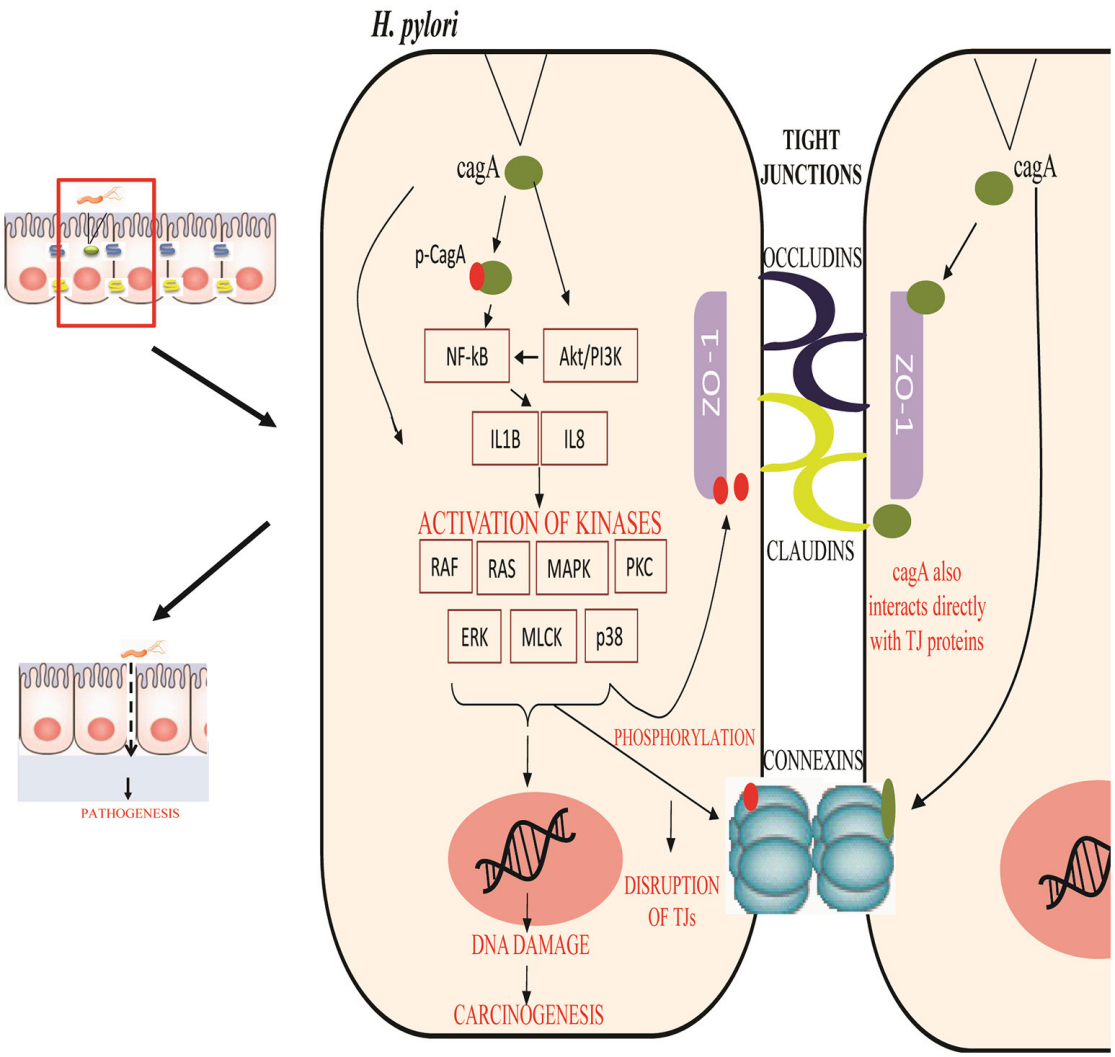
Regulation of tight junction permeability by *H. pylori*: Virulence factors of *H. pylori* CagA interact directly with junction proteins or modulate the signaling pathways to promote changes within their structure or function. Phosphorylated and non-phosphorylated forms of CagA induce inflammation and activates specific kinases to phosphorylate junction proteins along with other induced modifications. This leads to the epithelial barrier disruption and aids the entry of pathogen within cell easily.

Various studies have been abstracted to design a pathway leading to some important PTMs responsible for modulating the activity of TJ proteins. The cell-cell intersection is sealed by the members of the claudin family, whose extracellular loops connect the transmembrane domains thus forming the paracellular barrier. The C-terminal of claudin binds to the zonula occludens through their PDZ domains to seal the TJ (Nomme et al., 2015). Dysregulation of these proteins along with another gap junction protein CX32 expressed in gastric mucosa has been reported in delayed healing of gastric ulcer thus leading to gastritis or carcinoma (Wang et al., 2015). Activation or deactivation of these proteins under various stimuli is studied in this study. In the HPN model, each of these proteins is represented by a continuous entity activated by the flow of tokens passing through a series of transitions. Once *H. pylorus breaches* the gastric mucousal lining, it injects CagA through the type IV secretion system into epithelial cells leading to cell elongation and scattering. CagA has already been reported to mimic host cells by inhibiting kinase activities to elicits junctional and polarity defects (Amieva et al., 2003; Nishikawa et al., 2016). Kinases responsible for PTMs, especially for phosphorylation of TJ proteins, when disrupted by CagA lead to leaky gut barrier, thus aiding bacterial invasion. CagA works within host cell in both phosphorylated and non-phosphorylated forms to mediate pathogenicity. *H. pylori*-induced mutagenesis takes advantage of enhanced NF-κB in inflammation-associated carcinogenesis (Chiba et al., 2008) which modulates various cell functions majorly activation of IL-1 β and IL-8 (Noach et al., 1994; Maeda et al., 2000; Ferreira et al., 2016; Kameoka et al., 2016). The biological effects on activation on these inflammatory cytokines lead to the recruitment and activation of kinases favoring the phosphorylation-dependent disruption of TJ proteins (Figure 3). Other major mediators of *H. pylori-*induced pathogenesis include Protein kinase C (PKC) (Tohidpour, 2016), mitogen-activated protein kinase (MAPK) (Ding et al., 2008), p38, extracellular signal-regulated kinases (ERK) (Seo et al., 2013), and myosin light chain (MLC) (Khan et al., 2015). Interplay of these modulators has been shown in the designed model and thus their overall inhibitory effect on TJ proteins has been revealed.

## Results

### Sequence and structural features of crucial tight junction proteins: CLDN2, CX32, and ZO1

CLDN2, CX32, and ZO-1 protein sequences retrieved from SWISS-PROT have average lengths of 230, 283, and 1,748 amino acids, respectively. Sequence homologs of CLDN2, CX32 and ZO-1 from *Mus musculus, Rattus norvegicus, Canis familiaris, Cavia porcellus* and *Bos taurus* were aligned to find the conservation status. Results of alignment from CLC workbench (Supplementary Figure 1) revealed that all three proteins are well conserved among vertebrates. The conserved blocks were later checked for the sites having potential for epigenetic changes via PTMs (Supplementary Table 1).

**Figure 4:**
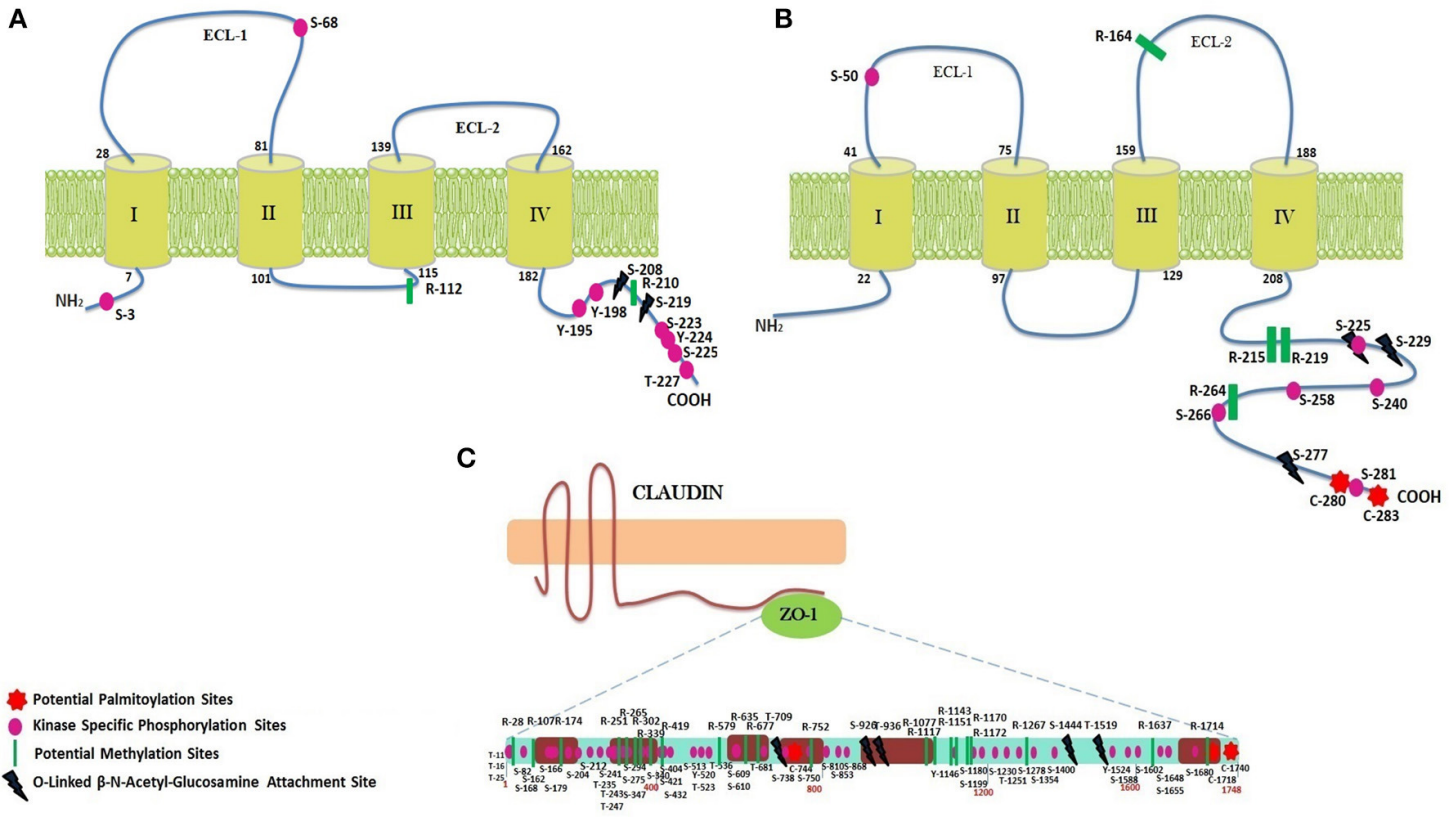
PTM sites identified within CLDN2, CX32, and ZO-1. **(A)** CLDN2 has two ECLs, one intracellular loop, four transmembrane domains, a short N-terminal, and a tail at C-terminus. Important PTM sites have been shown at their particular locations. **(B)** Structure of Cx32 (two ECLs, one intracellular loop, four transmembrane domains, a short N-terminal, and a tail at C-terminus) and predicted PTM sites at their particular locations have been shown. **(C)** ZO-1 has three PDZ domains, one SH3 and GK domain, and a long proline rich tail.

Predicted secondary structures of CLDN2, CX32 and ZO-1 help to determine the presence of transmembrane domains and loops at particular locations. CLDN2 and CX32 have four trans-membrane domains and two extracellular loops along with cytosolic N- and C-terminals (Figure 4) The adapter protein ZO-1 binds with TJ proteins at cytoplasmic side and is targeted by various transcription factors to modulate cell growth and permeability. It has three PDZ domains, a Src Homology-3 (SH3) domain and guanylate kinase (GK) domain along with a unique ZU5 domain at the C-terminal which is not possessed by other members of zona occludens' family (Haskins et al., 1998). Structural features of these proteins along with the predicted potential PTM sites have been shown in Figure 4.

### Methylation and palmitoylation sites exploited by pathogen to mediate signaling pathways

Methylation sites identified within TJ proteins may be targeted for therapeutic interventions in diseased condition. In CLDN2, two arginine residues at location 112 and 210 are estimated to be potential methylation sites using PMes program (Figure 4). These sites are highly conserved and located within intracellular cytoplasmic loop, thus can easily alter protein binding and loop conformations. Within CX32, five sites (R: 164, 215, 219, 223 and 264) showed potential for methylation. All sites are highly conserved except R-223, therefore excluded from further analysis. In ZO-1, 22 conserved arginine residues (28, 107,174, 251, 265, 302, 339, 341, 419, 579, 635, 677, 752, 1,077, 1,117, 1,143, 1,151, 1,170, 1,172, 1,210, 1,267, 1,637, and 1,714) were found to be potential methylation sites.

CLDN2 did not reveal any palmitoylation site whereas CX32 was found to have two cysteine residues (280 and 283) as potential palmitoylation sites (Figure 4). Both sites are located at the C-terminal of protein and remained 100% conserved among vertebrates that might affect the gap junction function by modulating the assembly, trafficking, disassembly and degradation of protein. In ZO-1, three sites (C: 744, 1,718 and 1,740) were predicted as conserved palmitoylation sites.

### Dynamics of the interplay amongst O-linked glycosylation and Yin-Yang sites reveal their role in *H. pylori* induced infection

Employing neural network based tools sites within CLDN2, CX32, and ZO-1, which can be targeted by O-linked glycosylation, were predicted. Only 2 exposed sites (Ser at 208 and 219) (Figure 4) were predicted within CLDN2 having potential to be an O-(beta)-GlcNAc site, among which S-219 showed high potential for O-linked glycosylation. These 2 sites also came out to be Yin-Yang sites also.

In CX32, five sites (T: 176, 269, and S: 225, 229, 277) showed their potential for O-GlcNAc, among them, three (S: 225, 229, and 277) had equal potential for phosphorylation thus designating them as Yin-Yang sites. O-GlcNAc sites of CX32 are also found to be highly conserved among other vertebrates. In ZO-1, 48 Ser, and Thr sites have potential for O-linked beta-glycosylation and among them 28 were found to be Yin-Yang sites (Supplementary Table 1). However, only five sites locating at reside number 709, 926, 936, 1,444, and 1,519 (Figure 4) were exposed and highly conserved among other species.

### Mapping evolutionary conserved kinase-specific phosphorylation sites having potential to augment disease progression

The possible Ser, Thr, and Tyr residues having potential to be phosphorylated were predicted among CLDN2, CX32, and ZO-1. 28 phosphorylation sites were predicted in CLDN2 and when analyzed for their location, only 12 sites being exposed were prioritized, as they are easily accessible to kinases. Among predicted sites only 9 sites (Ser: 3, 68, 192, 223, 225 Tyr: 195,198, 224, and Thr at 227) have potential for kinase specific phosphorylation (Figure 4). Another important aspect to consider these prioritized phospho-residues was their evolutionary conservation. All the exposed kinase specific phosphorylation sites are found to be highly conserved among vertebrates except Ser at position 192. In CX32, only Ser residues at position 50, 225, 229, 233, 240, 258, 266, and 281 are exposed and conserved kinase specific potential phosphorylation sites. Among these residues, Ser at position 229 and 233 are experimentally validated, verified by Phospho.ELM database (Diella et al., 2004) and S240 has already been predicted in a study to be a potential phosphorylation site (Locke et al., 2006). The other five sites are novel and needs to be evaluated experimentally. ZO-1, being a large protein, possess 167 residues capable to undergo kinase specific phosphorylation but only 76 residues were found to be novel and 100% conserved among vertebrates (Supplementary Table 1).

### Mathematical model reveals imperative route adapted by *H. pylori* to induce epigenetic changes within host TJ proteins

**Figure 5:**
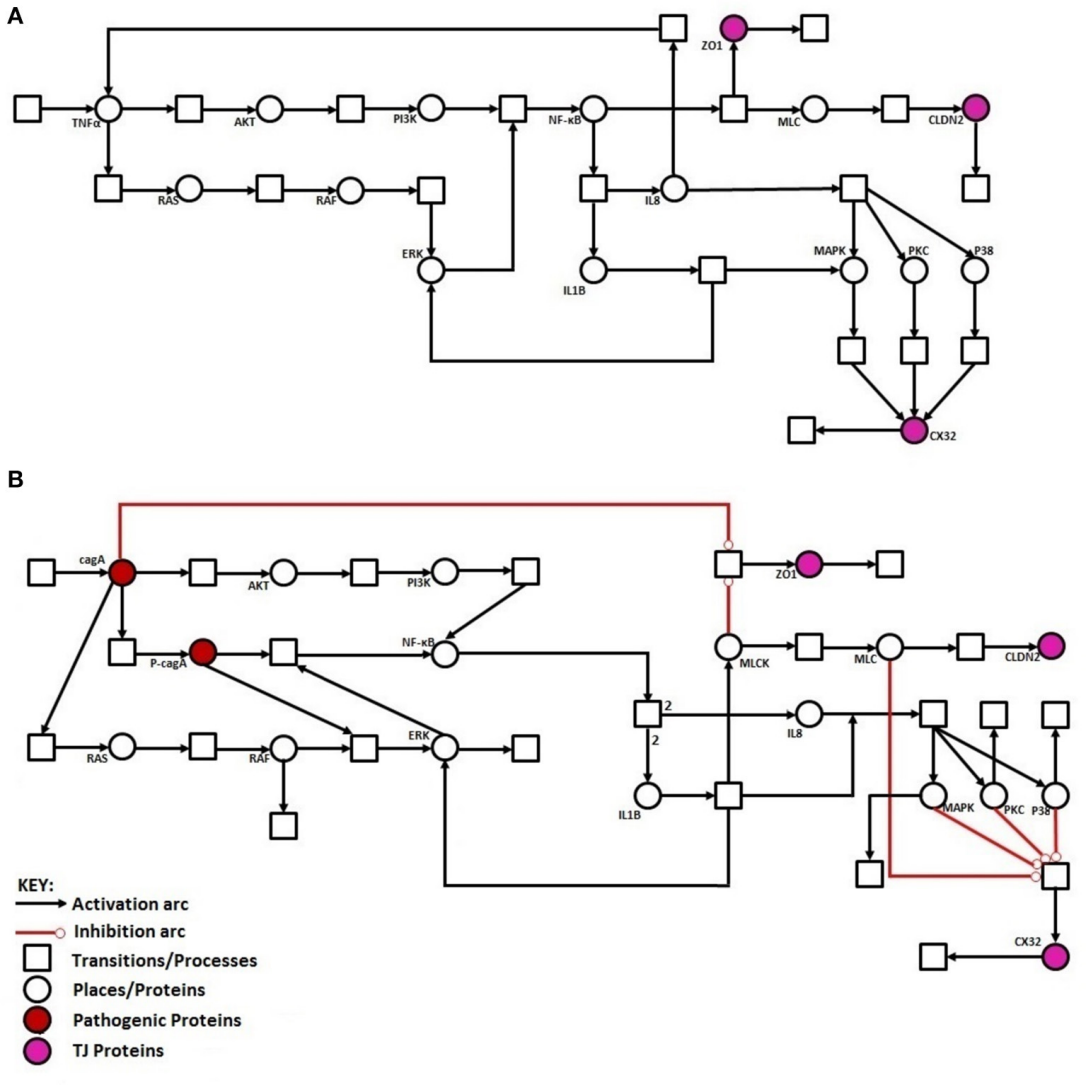
Illustration of proposed HPN models before and after *H. pylori* infection: **(A)** HPN model representing the normal behavior of proteins before infection. (**B)** HPN model representing the activation or deactivation of proteins/kinases after infection.

**Figure 6:**
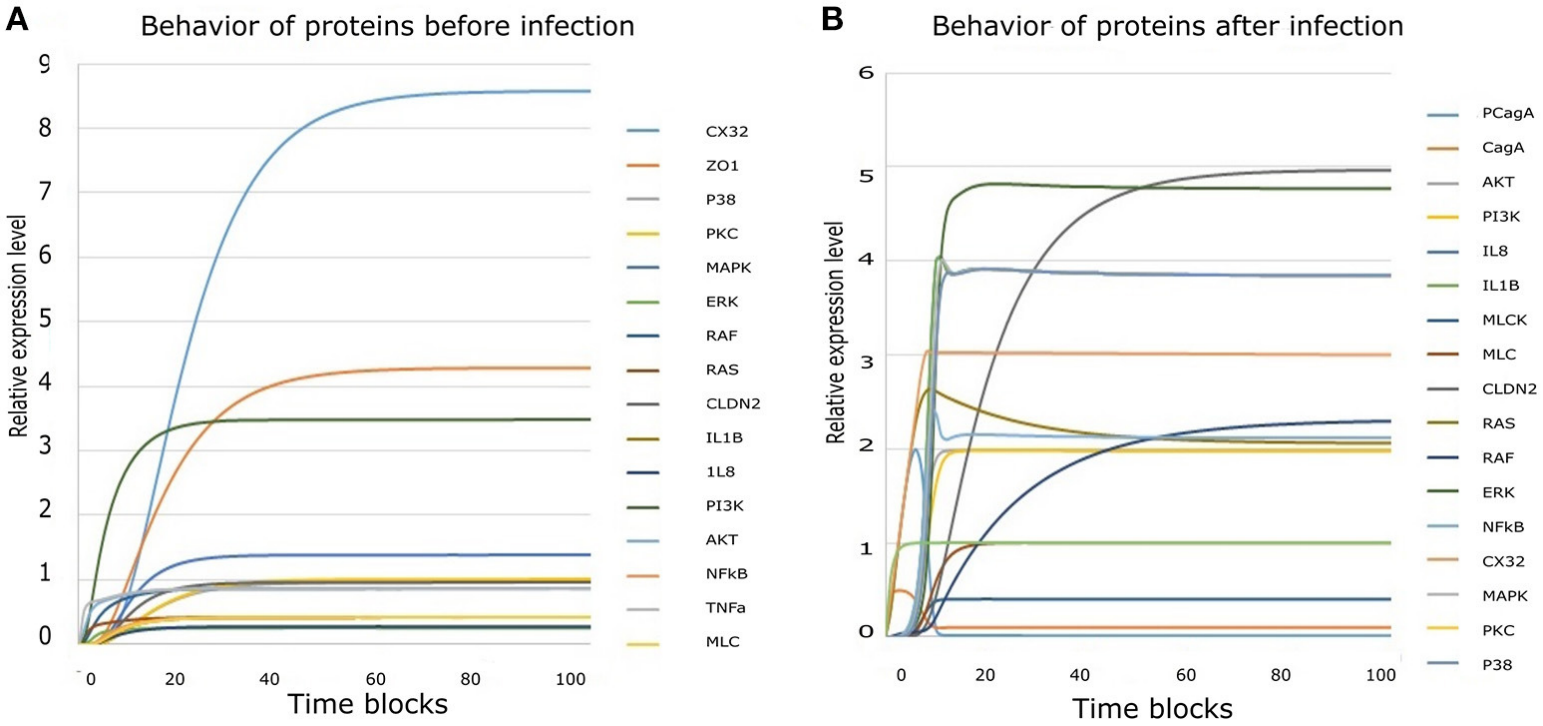
Simulation results derived from HPN model: **(A)** Simulation results of all entities showing their behavior before infection. **(B)** Collective simulation results of all entities after infection. x-axis represents time blocks which can be mins/hrs/days depending on the nature of experiment and y-axis represents relative expression level of proteins. Simulations were executed for 100 time blocks with the refresh rate of 5,000ms and 500 runs.

Regulation of TJ proteins has been modeled using HPNs as shown in Figures 5A,B. The changes in the behavior of some important regulatory entities after *H. pylori* infection and difference in their relative levels (expression/concentration) have been observed through step-wise simulations experiments. Simulations were executed for 100 time blocks with the refresh rate of 5,000 ms and 500 runs (Figures 6A,B). Currently, our model is truly qualitative and the rates of reactions are assumed by applying deductive reasoning (explained in Supplementary Data File 1) on the basis of biological role and interactions of proteins during normal and diseased conditions, affecting the expression of other proteins in the network (Supplementary Table 2 and Supplementary Data file 2). There are total 19 transitions (t0-t18) and 16 places in the normal model, whereas, 25 transitions (t0–t24) and 18 places in infection model (Figures 5A,B). The analysis and results of the simulations are discussed accordingly:

#### Activation of inflammatory cytokines induced after infection

**Figure 7:**
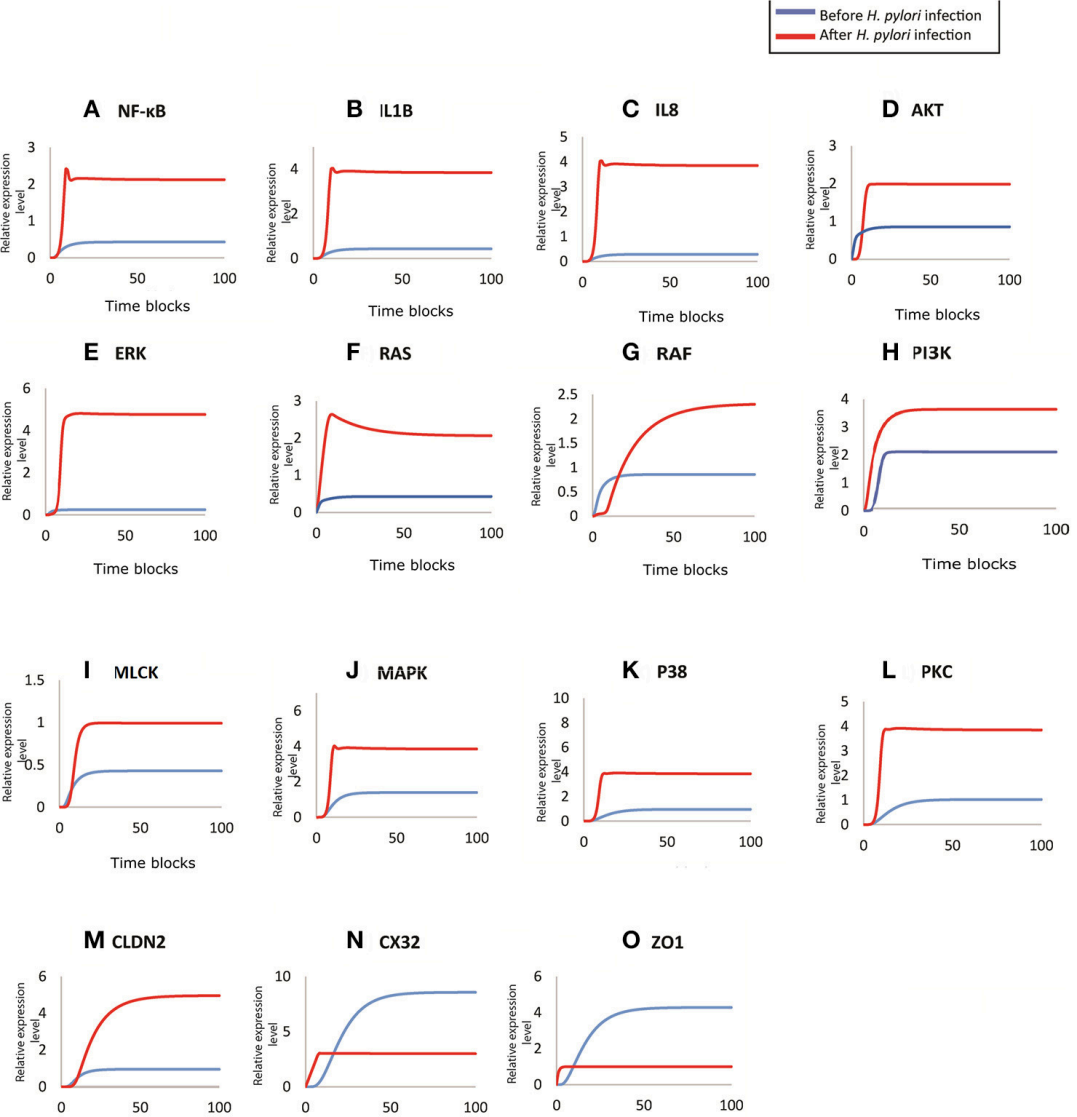
Comparison of relative changes in the response of entities before and after infection. Relative expression levels of **(A)** NF-kB, **(B)** IL1B, **(C)** IL8, **(D)** AKT, **(E)** ERK, **(F)** RAS, **(G)** RAF, **(H)** PI3K, **(I)** MLCK, **(J)** MAPK, **(K)** P38, **(L)** PKC, **(M)** CLDN2, **(N)** CX32, and **(O)** ZO1 have been shown on y-axis, whereas, the x-axis shows 100 time blocks. Entities are simulated after 500 runs with the refresh rate of 5,000 ms, in both models. Blue curve shown the activity level before infection and red curve represents the activity level of proteins after infection.

During *H. pylori* infection, NF-κB is activated rapidly to induce inflammation and the binding to transcription factors sites also regulates the functional opening of TJs (Ma et al., 2004). Simulation results of NF-κB obtained through designed HPN has been shown in Figure 7A, which clearly shows that before infection the expression level is low, as shown in blue curve. As infection persists within host cells, NF-κB expression elevates up to 2-folds as shown in the Figure 7A (red curve). The model also shows almost 2-fold increase in the production of NF-κB after infection (Figure 7A) which then leads to the activation of IL1β and IL8. Through our model, we evaluated this situation during infection, where production of both IL1β and IL8 increases up to 4 times than that of normal (Figures 7B,C), thus leading to inflammation induced epigenetic changes for the development of gastric ulcer and carcinoma. Graphs generated by HPN simulations depict the elevated behavior of IL1β and IL8 in such a manner that after infection there is a sudden increase in the production of both cytokines and a stable production throughout the infection is observed which may leading to inflammatory response in the real time.

#### Over-expression of kinases after *H. pylori* infection dysregulate other proteins and contribute toward pathogenesis

Targeted proteins during *H. pylori* infection mainly include ERK, AKT (Figure 7D), Ras-Raf (Figures 7F,G), MAPK (Figure 7J), MLCK (Figure 7I), p38 (Figure 7K), PI3K (Figure 7H) and PKC (Figure 7L). Non-phosphorylated CagA stimulated the production of Ras and Raf (appx. 2-fold) that in turn induces ERK production. whereas, phosphorylated CagA also stimulates ERK resulting up to 5-fold increase in its production during *H. pylori* infection (inferred from PN model - Figure 7E), leading to chronic inflammation. After infection, MLC concentration almost doubles as shown by red curve in the graph (Figure 7I) as compared to the normal conditions shown by blue curve. Such an elevated level of MLC in gut barriers subsequently results in barrier dysfunction aiding bacterial translocation across damaged epithelial lining. Furthermore, in response to cytokine production, overexpression of IL8 and IL1β also elevates the MAPK and p38 approximately four times than the normal cells (Figures 7J,K). An increased level of these kinases lead to altered cell proliferation (down regulation of TJ proteins such as CX32), cell survival rate and apoptosis.

#### Dysregulation of TJ proteins leading to leaky epithelial barrier

CagA directly attacks ZO-1 to attenuate its integrity thus altering the cell polarity (Ashida et al., 2012). Whereas, activation of MLCK also leads to dysregulation of ZO-1, thus exposing the basolateral surface (Yu et al., 2010). Almost 3-fold decrease has been observed in ZO-1 concentration at epithelial barriers after being infected by *H. pylori* (simulated results). As seen in Figure 7O, before infection, ZO-1 shows a gradual increase in its production (shown by blue curve) which stabilizes at a certain level to maintain its production necessary for cell integrity. Whereas, after infection, red curve shows suppressed expression of ZO-1, which clarify the outcome of pathogenic mechanism to disrupt TJ barrier function.

*H. pylori* infection induces hyper-methylation and other modifications within CX32 promoter and protein which suppresses the gene transcription (Wang et al., 2014) and reduces its expression. Simulation results of CX32 expression, as shown in Figure 7N, depicts a clear difference between normal and pathological conditions, where blue curve indicating state before infection shows almost 6 times more expression level as compared to the diseased condition (shown by red curve). HPN model also depicts almost 4-fold increase in the expression of CLDN2 in infected cells (shown by red curve in Figure 7M) as compared to normal physiological conditions (shown by blue curve).

## Discussion

*H. pylori* has evolved a wide range of stratagems to colonize and invade the distal parts of the stomach to induce diverse gastric pathologies, ranging from chronic gastritis and ulceration to neoplastic changes in the stomach (Wroblewski et al., 2009). Many studies have revealed some mechanisms adapted by the pathogen to affect host cells. In our study, we have focused on TJ disruption mediated by *H. pylori* infection. Theories explaining this phenomenon are yet not able to derive a consensus on the exact mechanism adapted by the pathogen (Shin et al., 2006; Al-Sadi et al., 2009; Wardill et al., 2014). The major target of *H. pylori* virulence factors are some specific host proteins (especially kinases), which can be stimulated to introduce site directed PTMs within TJ proteins. PTMs have potential to modulate and stimulate many life processes that is why they are hijacked by pathogens to strengthen their localization within host cells. It can be achieved by various ways such as phosphorylation, methylation, glycosylation, palmitoylation or modifications of amino acid side chains. Although some bacteria and virus attacks cell extracellularly but some pathogens invade the host cell to take refuge and escape immune response. To facilitate their entry and survival within host cells, effector proteins of pathogen stimulate some specific PTMs by modulating the host proteins thus targeting structural and regulatory barriers (Ribet and Cossart, 2010).

PTM sites identified within CLDN2, CX32, and ZO-1 in this study have highlighted some crucial and important sites which can be targeted by the pathogen to dysregulate the normal functioning of barrier proteins, thus defecting the cell polarity. These sites may be targeted for therapeutic interventions in diseased condition. All predicted sites are highly conserved, emphasizing on their evolutionary importance. Whereas, the location of these sites also play significant role in their targeted function. *H. pylori* infection has also been reported to be linked with regulation and dysregulation of genes, proteins and their promoters via various modifications, especially methylation, initiating gastric carcinoma (Parsonnet et al., 1991; Akhtar et al., 2001; Blaser and Berg, 2001; Perri et al., 2007). Their ability to induce methylation has also been confirmed within animal model (Niwa et al., 2010). Thus, we found it quite crucial to target important methylation sites within TJ proteins. Some methylation sites of TJ proteins lie within functional domains, thus, predicting their effective roles in maintaining the cell integrity. R164 predicted within ECL2 of CX32 can be of great importance as these loops are maintaining the cell integrity by docking with neighboring connexins. Whereas, other four predicted sites lying on C-terminal of protein may also trigger major functions, as tail of connexins have been reportedly involved in modulating gene expression and cell-cycle control via binding proteins (Giepmans, 2004). Similarly, R-28 within PDZ1 domain and R-251 in PDZ2 domain of ZO-1 might be of great interest as these domains are responsible for anchoring ZO-1 with claudins and connexins (Thévenin et al., 2013). R-579 lies within Src Homology-3 (SH3) domain much important for protein interactions. Modification of residues in this domain may lead to disrupted protein-protein interactions, thus, modulating signaling pathways (Thévenin et al., 2013). R-635, 677 and 752 are within Guanylate kinase (GK) domain of ZO-1, where occludins and adherens junction proteins can bind.

Phosphorylation, one of the most common PTM is also triggered by pathogens to influence some significant pathways. *H. pylori* also targets TJ proteins for unwanted phosphorylation/dephosphorylation modifications to loosen the cell to cell junction. Effect of modification truly depends in number and location of PTM sites. Phosphorylation sites identified within CLDN2, CX32, and ZO-1 also revealed some crucial points that can be targeted by *H. pylori* during infection. Tyr-224 of CLDN2 has already been studied as potential phosphorylation site which modulates the binding of CLDN2 with ZO-1, as it has already been identified as key factor for regulating affinity between claudins and PDZ domain (Nomme et al., 2015). Presence of such a crucial site makes adjacent amino acids (S-223 and S-225) more significant to be tested experimentally as they have also been identified as potential curated phosphorylation sites by PhosphoSite Plus (Hornbeck et al., 2015) and also in our data set. As CLDN2 is involved in barrier leakiness and PTMs use to influence its pore forming ability, therefore, it should be systematically analyzed for its phosphorylation and other modification sites to unravel the mechanism of its regulation or dysregulation. Similarly, in CX32 Ser at position 229 and 233 are experimentally validated, verified by Phospho.ELM database (Diella et al., 2004) and S240 has already been predicted in a study to be a potential phosphorylation site (Locke et al., 2006). Whereas, other five sites may be novel and would needs to be evaluated experimentally. The prediction of experimentally validated residues among our data set validates the potential of phosphorylation for selected residues. Most of these phosphorylation sites harbor C-terminal of CX32 except Ser at 50th position that lies within ECL1. C-terminal of connexins is a major influential part for protein trafficking and studies have reported that phosphorylation of some amino acids at the tail can also alter the protein half-life and gap junction assembly (Johnstone et al., 2012). Sites having potential to be phosphorylated within ZO-1 are also found within crucial domains directly involved in the binding with occludins, important TJ proteins. Modification in such domains may lead to disassociation of ZO-1 from TJ proteins, hence, loosening the epithelial barriers and facilitate the entry of pathogen. *H. pylori* alters the expression of different kinases, proteins which catalyze phosphorylation events, to infer normal modifications through their virulence factors (Ribet and Cossart, 2010). All predicted phosphorylation sites when checked for their kinases, reveal some specific kinases triggered during infection. Kinases affected especially by *H. pylori* and its virulence factors were dug out through extensive literature search. The regulation in expression of these kinases by CagA are then modeled using HPNs, which presents the possible route adopted by CagA to induce PTMs in TJ proteins. The model designed in this study helps us to investigate the downstream effect of injected CagA within host cells under pathophysiological conditions. For now, we ignored the effect of other factors or proteins modulating the expression of *H. pylori* specific kinases. Our model was refined using experimental data and current knowledge of protein interactions and their influence on each other's expression both in normal and diseased states. One of the major key factor in the regulation of kinases is activation of cytokines IL1β and IL8. Production of these proinflammatory cytokines is one of the hallmarks of the gastric mucosa infection by *H. pylori* which plays significant role in disease progression. Usually CagA+ strains promote higher production of IL1β and IL8 resulting in an increased risk of peptic ulcer and gastric cancer (Noach et al., 1994; Harris et al., 1996; Nagashima et al., 2015; Ferreira et al., 2016). The increased level of IL1β and IL8 during infection is stimulated by both phosphorylated and unphosphorylated CagA, as it triggers the production of NF-κB to almost double. Elevated expression of NF-κB I turn activates various kinases like MLCK, MAPK, PKC, P38 etc., which induce phosphorylations at different sites of TJ proteins. Phosphorylation of MLC by myosin light chain kinase (MLCK) causes distension in TJs thus affecting the cell permeability. Increased IL1β also stimulates the activation of MLCK during *H. pylori* infection (Ashida et al., 2012). Phosphorylation and activation of these kinases thus induce specific PTMs within TJ proteins which help the pathogen in cellular vacuolation, loss of membrane integrity and apoptosis leading to gastric atrophy or intestinal metaplasia. Pattern of all these changes have been predicted through our model under the influence of CagA. Simulation results of this model (of disease condition) are validated by comparing it by modeling expression of these proteins under normal conditions. Simulation results of our proposed HPN models are in complete agreement with the experimental results (Table 1), thus verifying the predictions of our model.

In conclusion, our HPN model of intracellular signaling after *H. pylori* infection provides insight on the mechanism adopted by pathogen to induce PTMs within TJ proteins. Model clearly shows that, as a result of action of specific kinases, expression of CX32 and ZO-1 decreases up to significant levels whereas CLDN2 gets overexpressed to promote paracellular cation leak. Despite the limitation of being adapted from previous literature, our model reflects the sequence of events and captures the logical interactions among entities through various mechanisms. Such models can further be expanded to unveil the altered or adapted mechanisms by pathogen during or before the induction of infection and pathogenesis.

## Conclusion

We believe that *H. pylori* significantly modulate the host TJ proteins and their interactions to direct its entry within host epithelial cells via PTMs. To induce specific PTMs, pathogen disrupts the signaling pathways to express or suppress specific proteins and kinases leading to alteration within the barrier proteins. Specific PTM sites have been predicted within selected TJ proteins, these can be further targeted to infer their potential impact *in vitro* and *in vivo* models. The HPN model proposed in this study was interesting and revealed a possible mechanism adapted by the *H. pylori* to promote the leaky barrier. The results presented here are truly qualitative, hence need experimental validation, which can help to improve the model by inclusion of quantitative data. Similar approaches can also help to infer specific targets for specific interventions such as drugs and vaccines or their combinations to combat the *H. pylori* infection. The approach can be extended to other host-pathogen interactions model for understating the progression of diseases and prediction of potential therapeutic targets.

## Author contributions

AA and AN designed the study. AN contributed to analysis, interpretation of data and draft composition. AO, FA, and AI helped in designing mathematical models and proteins' analysis. AA, AO, FA, JA, and AI critically analyzed the draft and helped AN in organizing the final version.

### Conflict of interest statement

The authors declare that the research was conducted in the absence of any commercial or financial relationships that could be construed as a potential conflict of interest.
