# Supplementary material for: Petri Net-Based Model of Helicobacter pylori Mediated Disruption of Tight Junction Proteins in Stomach Lining during Gastric Carcinoma
Source: Front Microbiol. 2017 Sep 6;8:1682. doi: 10.3389/fmicb.2017.01682 (PMC5592237; doi:10.3389/fmicb.2017.01682)
Supplement: Supplementary file 1 [file Image1.PDF]

Supplementary Material

Petri Net-based model of *Helicobacter pylori* mediated disruption of tight junction proteins in stomach lining during gastric carcinoma

Anam Naz[1], Ayesha Obaid[1], Faryal Mehwish Awan[1], Aqsa Ikram[1], Jamil Ahmad[2], Amjad Ali[1*]

*** Correspondence:** Amjad Ali, amjaduni@gmail.com

1 Supplementary Figures

1.1 Supplementary Figure 1

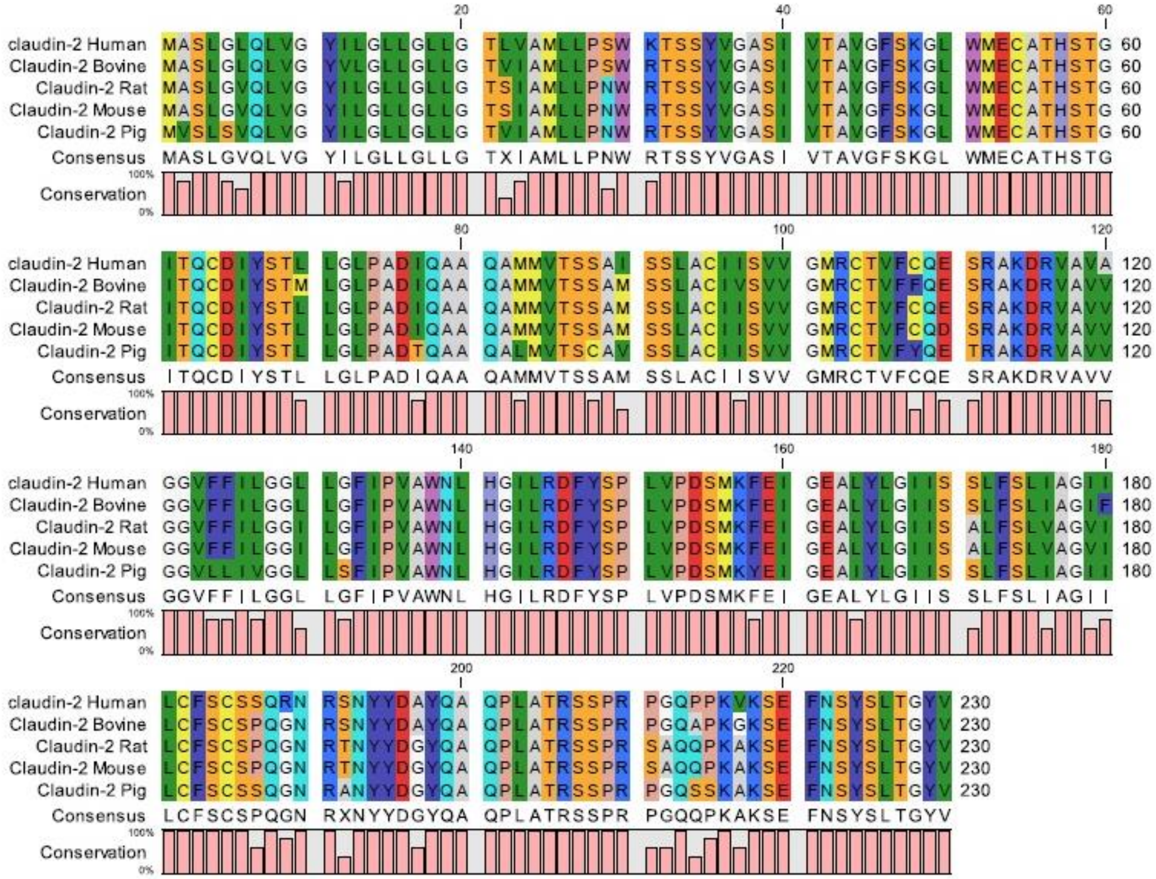

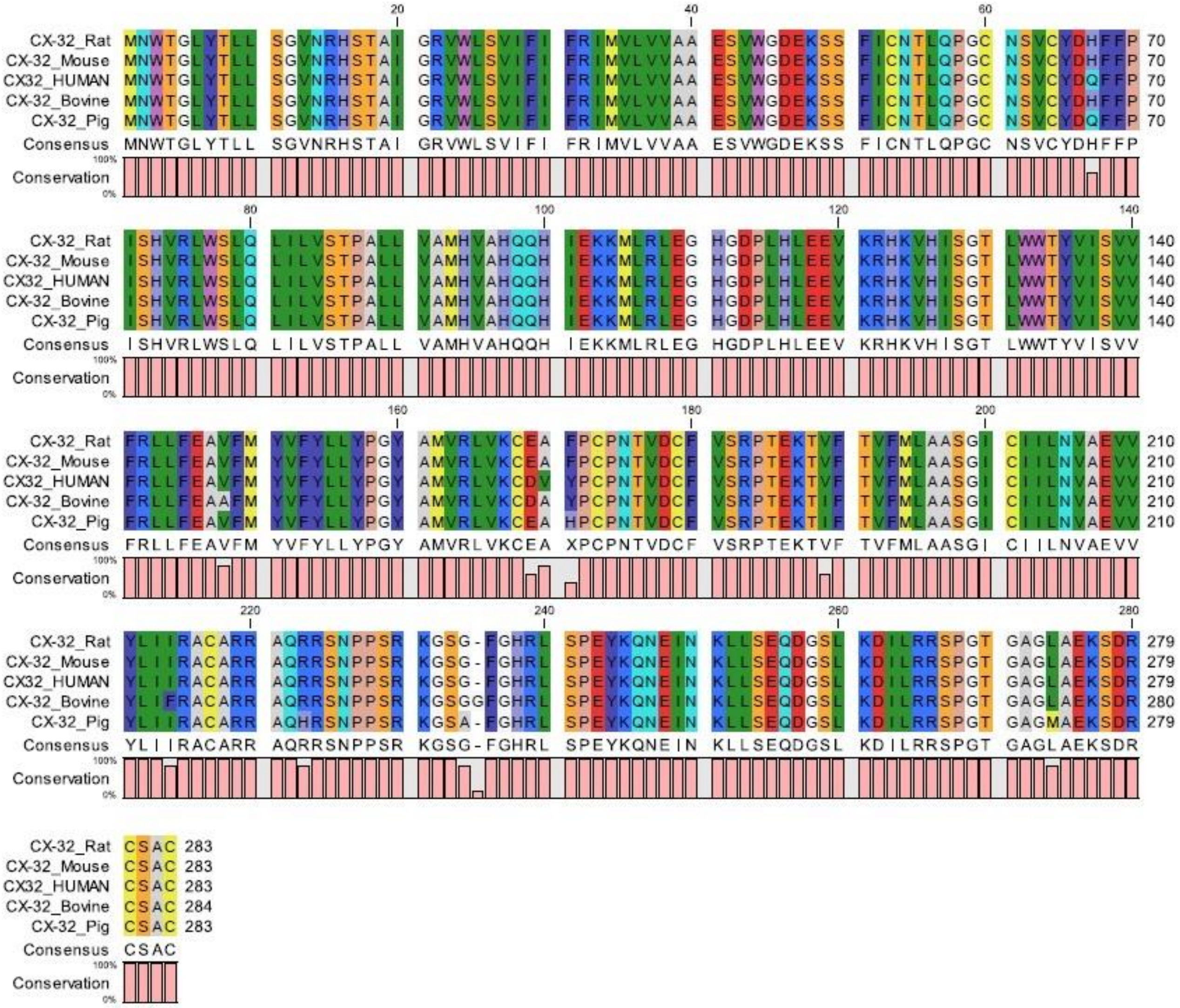

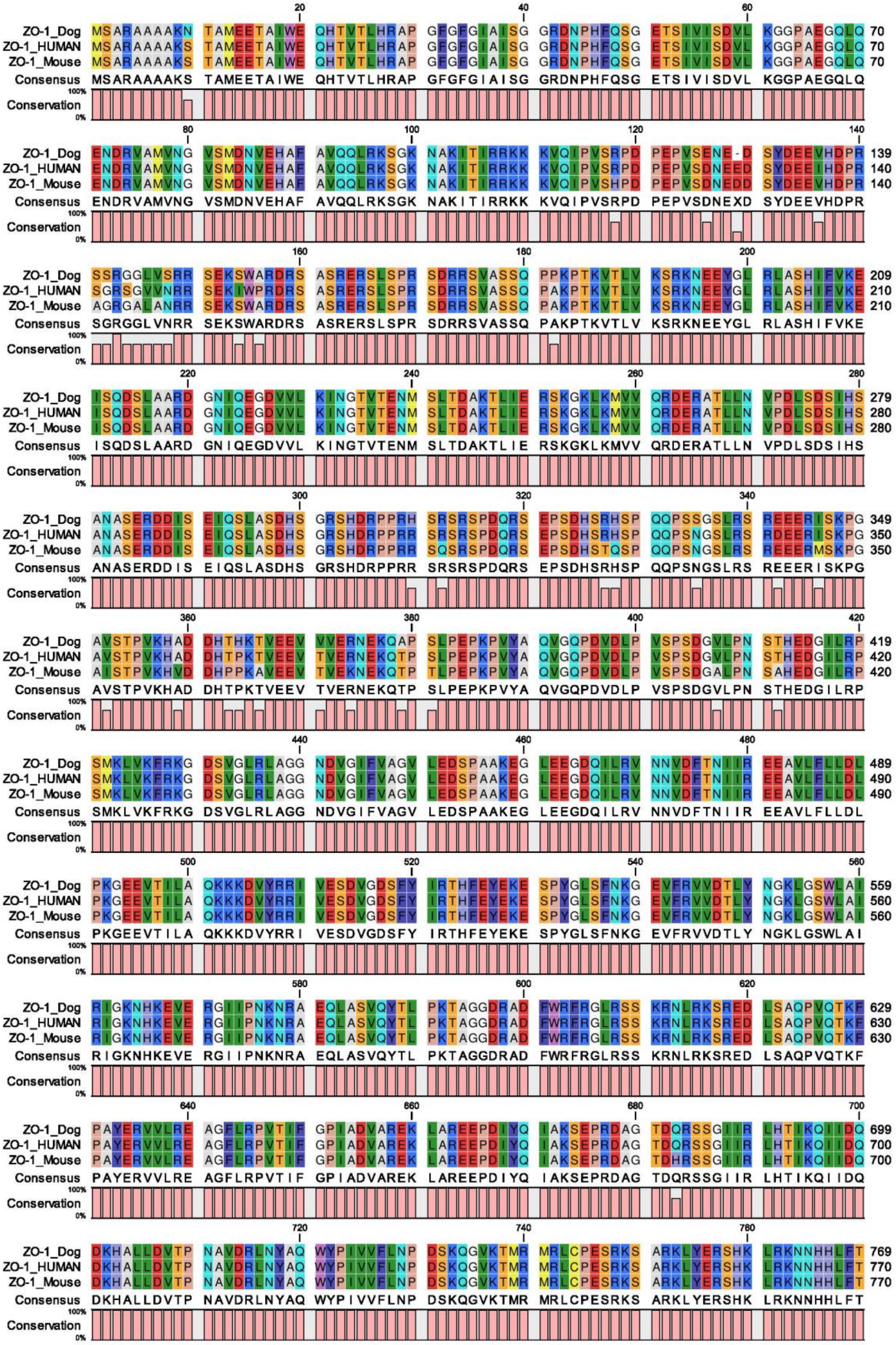
20 40 60
ZO-1_Dog MSARAAAAKN TAMEETAIWE QHTVTLHRAP GFGFGIAISG GRDNPHFQSG ETSIVISDVL KGGPAEGQLQ 70
ZO-1_HUMAN MSARAAAAKS TAMEETAIWE QHTVTLHRAP GFGFGIAISG GRDNPHFQSG ETSIVISDVL KGGPAEGQLQ 70
ZO-1_Mouse MSARAAAAKS TAMEETAIWE QHTVTLHRAP GFGFGIAISG GRDNPHFQSG ETSIVISDVL KGGPAEGQLQ 70
Consensus MSARAAAAKS TAMEETAIWE QHTVTLHRAP GFGFGIAISG GRDNPHFQSG ETSIVISDVL KGGPAEGQLQ
100%
Conservation
0%
80 100 120 140
ZO-1_Dog ENDRVAMVNG VSMDNVEHAF AVQQLRKSGK NAKITIRRKK KVQIPVSRPD PEPVSENE-D SYDEEVHDPR 139
ZO-1_HUMAN ENDRVAMVNG VSMDNVEHAF AVQQLRKSGK NAKITIRRKK KVQIPVSRPD PEPVSDNEED SYDEEIHDPR 140
ZO-1_Mouse ENDRVAMVNG VSMDNVEHAF AVQQLRKSGK NAKITIRRKK KVQIPVSHPD PEPVSDNEDD SYDEEVHDPR 140
Consensus ENDRVAMVNG VSMDNVEHAF AVQQLRKSGK NAKITIRRKK KVQIPVSRPD PEPVSDNEXD SYDEEVHDPR
100%
Conservation
0%
160 180 200
ZO-1_Dog SSRGGLVSRR SEKSWARDRS ASRERSLSPR SDRRSVASSQ PPKPTKVTLV KSRKNEEYGL RLASHIFVKE 209
ZO-1_HUMAN SGRSGVVNRR SEKIWPRDRS ASRERSLSPR SDRRSVASSQ PAKPTKVTLV KSRKNEEYGL RLASHIFVKE 210
ZO-1_Mouse AGRGALANRR SEKSWARDRS ASRERSLSPR SDRRSVASSQ PAKPTKVTLV KSRKNEEYGL RLASHIFVKE 210
Consensus SGRGGLVNRR SEKSWARDRS ASRERSLSPR SDRRSVASSQ PAKPTKVTLV KSRKNEEYGL RLASHIFVKE
100%
Conservation
0%
220 240 260 280
ZO-1_Dog ISQDSLAARD GNIQEGDVVL KINGTVTENM SLTDAKTLIE RSKGKLKMVV QRDERATLLN VPDLSDSIHS 279
ZO-1_HUMAN ISQDSLAARD GNIQEGDVVL KINGTVTENM SLTDAKTLIE RSKGKLKMVV QRDERATLLN VPDLSDSIHS 280
ZO-1_Mouse ISQDSLAARD GNIQEGDVVL KINGTVTENM SLTDAKTLIE RSKGKLKMVV QRDERATLLN VPDLSDSIHS 280
Consensus ISQDSLAARD GNIQEGDVVL KINGTVTENM SLTDAKTLIE RSKGKLKMVV QRDERATLLN VPDLSDSIHS
100%
Conservation
0%
300 320 340
ZO-1_Dog ANASERDDIS EIQSLASDHS GRSHDRPPRH SRSRSPDQRS EPSDHSRHSP QQPSSGSLRS REEERISKPG 349
ZO-1_HUMAN ANASERDDIS EIQSLASDHS GRSHDRPPRR SRSRSPDQRS EPSDHSRHSP QQPSNGSLRS RDEERISKPG 350
ZO-1_Mouse ANASERDDIS EIQSLASDHS GRSHDRPPRR SQSRSPDQRS EPSDHSTQSP QQPSNGSLRS REEERMSKPG 350
Consensus ANASERDDIS EIQSLASDHS GRSHDRPPRR SRSRSPDQRS EPSDHSRHSP QQPSNGSLRS REEERISKPG
100%
Conservation
0%
360 380 400 420
ZO-1_Dog AVSTPVKHAD DHTHKTVEEV VVERNEKQAP SLPEPKPVYA QVGQPDVDLP VSPSDGVLPN STHEDGILRP 419
ZO-1_HUMAN AVSTPVKHAD DHTPKTVEEV TVERNEKQTP SLPEPKPVYA QVGQPDVDLP VSPSDGVLPN STHEDGILRP 420
ZO-1_Mouse AISTPVKHVD DHPPKAVEEV TVEKNEKQTP TLPEPKPVYA QVGQPDVDLP VSPSDGALPN SAHEDGILRP 420
Consensus AVSTPVKHAD DHTPKTVEEV TVERNEKQTP SLPEPKPVYA QVGQPDVDLP VSPSDGVLPN STHEDGILRP
100%
Conservation
0%
440 460 480
ZO-1_Dog SMKLVKFRKG DSVGLRLAGG NDVGIFVAGV LEDSPAAKEG LEEGDQILRV NNVDFTNIIR EEAVLFLLDL 489
ZO-1_HUMAN SMKLVKFRKG DSVGLRLAGG NDVGIFVAGV LEDSPAAKEG LEEGDQILRV NNVDFTNIIR EEAVLFLLDL 490
ZO-1_Mouse SMKLVKFRKG DSVGLRLAGG NDVGIFVAGV LEDSPAAKEG LEEGDQILRV NNVDFTNIIR EEAVLFLLDL 490
Consensus SMKLVKFRKG DSVGLRLAGG NDVGIFVAGV LEDSPAAKEG LEEGDQILRV NNVDFTNIIR EEAVLFLLDL
100%
Conservation
0%
500 520 540 560
ZO-1_Dog PKGEEVTILA QKKKDVYRRI VESDVGDSFY IRTHFEYEKE SPYGLSFNKG EVFRVVDTLY NGKLGSWLAI 559
ZO-1_HUMAN PKGEEVTILA QKKKDVYRRI VESDVGDSFY IRTHFEYEKE SPYGLSFNKG EVFRVVDTLY NGKLGSWLAI 560
ZO-1_Mouse PKGEEVTILA QKKKDVYRRI VESDVGDSFY IRTHFEYEKE SPYGLSFNKG EVFRVVDTLY NGKLGSWLAI 560
Consensus PKGEEVTILA QKKKDVYRRI VESDVGDSFY IRTHFEYEKE SPYGLSFNKG EVFRVVDTLY NGKLGSWLAI
100%
Conservation
0%
580 600 620
ZO-1_Dog RIGKNHKEVE RGIIPNKNRA EQLASVQYTL PKTAGGDRAD FWRFRGLRSS KRNLRKSRED LSAQPVQTKF 629
ZO-1_HUMAN RIGKNHKEVE RGIIPNKNRA EQLASVQYTL PKTAGGDRAD FWRFRGLRSS KRNLRKSRED LSAQPVQTKF 630
ZO-1_Mouse RIGKNHKEVE RGIIPNKNRA EQLASVQYTL PKTAGGDRAD FWRFRGLRSS KRNLRKSRED LSAQPVQTKF 630
Consensus RIGKNHKEVE RGIIPNKNRA EQLASVQYTL PKTAGGDRAD FWRFRGLRSS KRNLRKSRED LSAQPVQTKF
100%
Conservation
0%
640 660 680 700
ZO-1_Dog PAYERVVLRE AGFLRPVTIF GPIADVAREK LAREEPDIYQ IAKSEPRDAG TDQRSSGIIR LHTIKQIIDQ 699
ZO-1_HUMAN PAYERVVLRE AGFLRPVTIF GPIADVAREK LAREEPDIYQ IAKSEPRDAG TDQRSSGIIR LHTIKQIIDQ 700
ZO-1_Mouse PAYERVVLRE AGFLRPVTIF GPIADVAREK LAREEPDIYQ IAKSEPRDAG TDHRSSGIIR LHTIKQIIDQ 700
Consensus PAYERVVLRE AGFLRPVTIF GPIADVAREK LAREEPDIYQ IAKSEPRDAG TDQRSSGIIR LHTIKQIIDQ
100%
Conservation
0%
720 740 760
ZO-1_Dog DKHALLDVTP NAVDRLNYAQ WYPIVVFLNP DSKQGVKTMR MRLCPESRKS ARKLYERSHK LRKNNHHLFT 769
ZO-1_HUMAN DKHALLDVTP NAVDRLNYAQ WYPIVVFLNP DSKQGVKTMR MRLCPESRKS ARKLYERSHK LRKNNHHLFT 770
ZO-1_Mouse DKHALLDVTP NAVDRLNYAQ WYPIVVFLNP DSKQGVKTMR MRLCPESRKS ARKLYERSHK LRKNNHHLFT 770
Consensus DKHALLDVTP NAVDRLNYAQ WYPIVVFLNP DSKQGVKTMR MRLCPESRKS ARKLYERSHK LRKNNHHLFT
100%
Conservation
0%

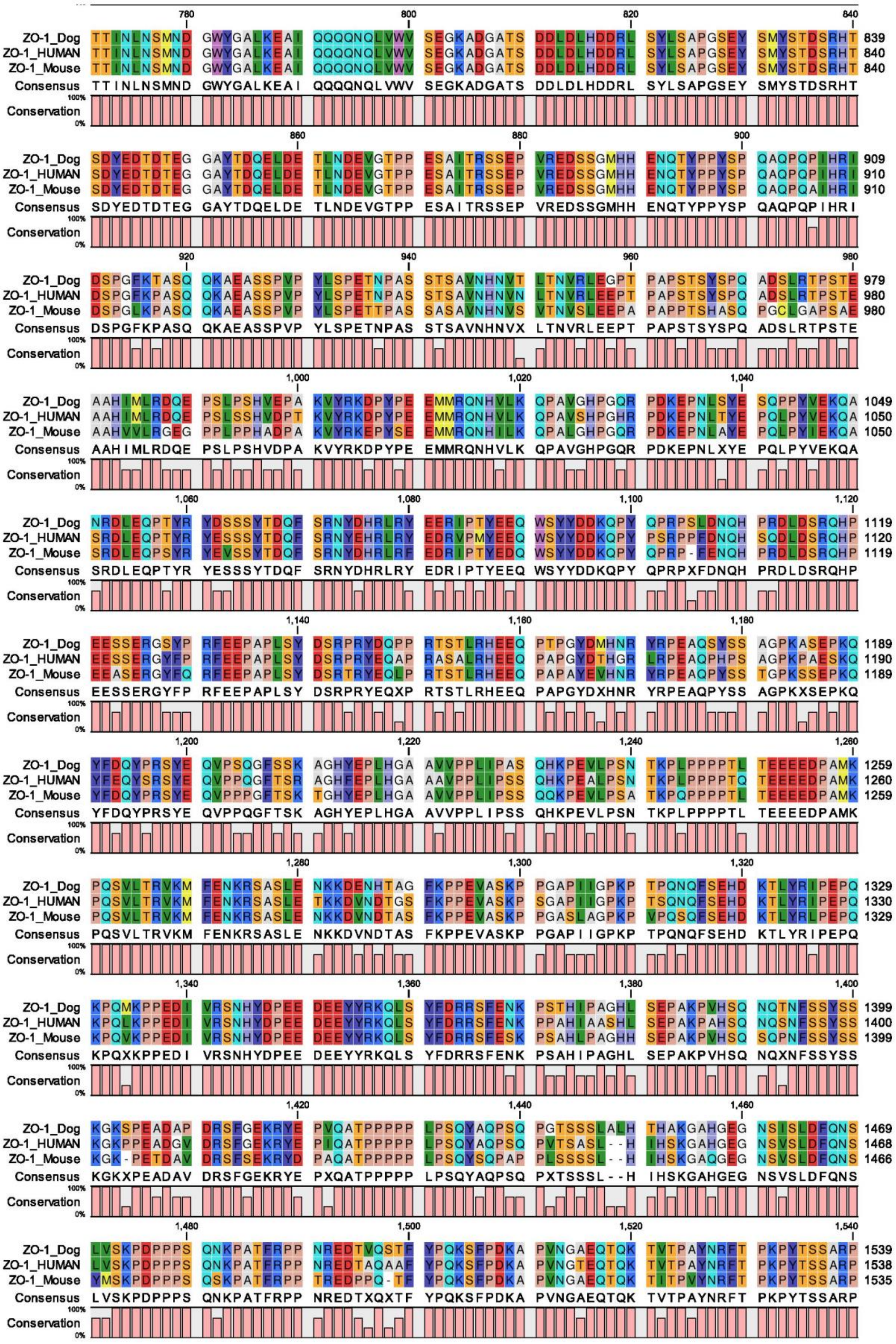
ZO-1_Dog
ZO-1_HUMAN
ZO-1_Mouse
Consensus
Conservation
TTINLNSMND GWYGALKEAI QQQQNQLVWV SEGKADGATS DDLDLHDDRL SYLSAPGSEY SMYSTDSRHT
SDYEDTDTEG GAYTDQELDE TLNDEVGTPP ESAITRSSEP VREDSSGMHH ENQTYPPYSP QAQPQPIHRI
DSPGFKPASQ QKAEASSPVP YLSPETNPAS STSAVNHNVX LTNVRLEEPT PAPSTSYSPQ ADSLRTPSTE
AAHIMLRDQE PSLPSHVDPA KVYRKDPYPE EMMRQNHVLK QPAVGHPGQR PDKEPNLXYE PQLPYVEKQA
SRDLEQPTYR YESSSYTDQF SRNYDHRLRY EDRIPTYEEQ WSYYDDKQPY QPRPXFDNQH PRDLDSRQHP
EESSERGYFP RFEEPAPLSY DSRPRYEQXP RTSTLRHEEQ PAPGYDXHNR YRPEAQPYSS AGPKXSEPKQ
YFDQYPRSYE QVPPQGFTSK AGHYEPLHGA AVVPPLIPSS QHKPEVLPSN TKPLPPPPTL TEEEEDPAMK
PQSVLTRVKM FENKRSASLE NKKDVNDTAS FKPPEVASKP PGAPIIGPKP TPQNQFSEHD KTLYRIPEPQ
KPQXKPPEDI VRSNHYDPEE DEEYYRKQLS YFDRRSFENK PSAHIPAGHL SEPAKPVHSQ NQXNFSSYSS
KGKXPEADAV DRSFGEKRYE PXQATPPPPP LPSQYAQPSQ PXTSSSL--H IHSKGAHGEG NSVSLDFQNS
LVSKPDPPPS QNKPATFRPP NREDTXQXTF YPQKSFPDKA PVNGAEQTQK TVTPAYNRFT PKPYTSSARP

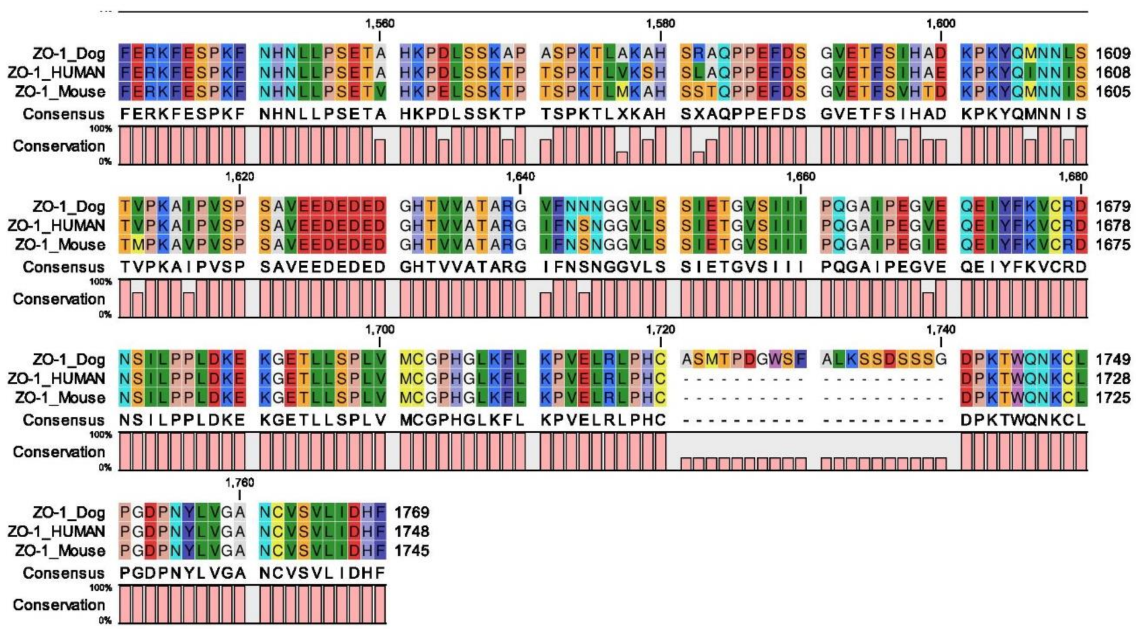

Supplementary Figure 1.. Alignments of CLDN2, CX32 and ZO-1 proteins to reveal conserved regions.
