# Supplementary material for: Petri Net-Based Model of Helicobacter pylori Mediated Disruption of Tight Junction Proteins in Stomach Lining during Gastric Carcinoma
Source: Front Microbiol. 2017 Sep 6;8:1682. doi: 10.3389/fmicb.2017.01682 (PMC5592237; doi:10.3389/fmicb.2017.01682)
Supplement: Supplementary file 2 [file Table1.docx]

Supplementary Material

Petri Net-based model of *Helicobacter pylori* mediated disruption of tight junction proteins in stomach lining during gastric carcinoma

Anam Naz^1^, Ayesha Obaid^1^, Faryal Mehwish Awan^1^, Aqsa Ikram^1^, Jamil Ahmad^2^, Amjad Ali^1*^

*** Correspondence:** Amjad Ali, amjaduni@gmail.com

# Supplementary Table 1

**Supplementary Table 1:** PTM sited identified within CLDN2, CX32 and ZO-1

| O-GlcNAc Sites | | | |
| --- | --- | --- | --- |
|  | Amino acid | Position | Conservation |
| Claudin-2 | S | 208 | 100% |
|  | S | 219 | 100% |
| Connexin-32 | S | 225 | 100% |
|  | S | 229 | 100% |
|  | S | 277 | 100% |
| ZO-1 | T | 709 | 100% |
|  | S | 926 | 100% |
|  | T | 936 | 100% |
|  | S | 1444 | 100% |
|  | T | 1519 | 100% |
| Palmitoylation Sites | | | |
|  | Amino acid | Position | Conservation |
| Claudin-2 | No site! |  |  |
| Connexin-32 | C | 280 | 100% |
|  | C | 283 | 100% |
| ZO-1 | C | 744 | 100% |
|  | C | 1718 | 100% |
|  | C | 1740 | 100% |
| Methylation Sites | | | |
|  | Amino acid | Position | Conservation |
| Claudin-2 | R | 112 | 100% |
|  | R | 210 | 100% |
| Connexin-32 | R | 164 | 100% |
|  | R | 215 | 100% |
|  | R | 219 | 100% |
|  | R | 264 | 100% |
| ZO-1 | R | 28 | 100% |
|  | R | 107 | 100% |
|  | R | 174 | 100% |
|  | R | 251 | 100% |
|  | R | 265 | 100% |
|  | R | 302 | 100% |
|  | R | 339 | 100% |
|  | R | 341 | 100% |
|  | R | 419 | 100% |
|  | R | 579 | 100% |
|  | R | 635 | 100% |
|  | R | 677 | 100% |
|  | R | 752 | 100% |
|  | R | 1077 | 100% |
|  | R | 1117 | 100% |
|  | R | 1143 | 100% |
|  | R | 1151 | 100% |
|  | R | 1170 | 100% |
|  | R | 1172 | 100% |
|  | R | 1267 | 100% |
|  | R | 1637 | 100% |
|  | R | 1714 | 100% |
| Kinase Specific Phosphorylation Sites | | | |
|  | Amino acid | Position | Conservation |
| Claudin-2 | S | 3 | 100% |
|  | S | 68 | 100% |
|  | Y | 195 | 100% |
|  | Y | 198 | 100% |
|  | S | 223 | 100% |
|  | Y | 224 | 100% |
|  | S | 225 | 100% |
|  | T | 227 | 100% |
| Connexin-32 | S | 50 | 100% |
|  | S | 225 | 100% |
|  | S | 240 | 100% |
|  | S | 258 | 100% |
|  | S | 266 | 100% |
|  | S | 281 | 100% |
| ZO-1 | T | 11 | 100% |
|  | T | 16 | 100% |
|  | T | 25 | 100% |
|  | S | 82 | 100% |
|  | S | 162 | 100% |
|  | S | 166 | 100% |
|  | S | 168 | 100% |
|  | S | 179 | 100% |
|  | S | 204 | 100% |
|  | S | 212 | 100% |
|  | T | 235 | 100% |
|  | S | 241 | 100% |
|  | T | 243 | 100% |
|  | T | 247 | 100% |
|  | S | 275 | 100% |
|  | S | 294 | 100% |
|  | S | 315 | 100% |
|  | S | 340 | 100% |
|  | S | 347 | 100% |
|  | S | 404 | 100% |
|  | S | 411 | 100% |
|  | S | 421 | 100% |
|  | S | 432 | 100% |
|  | S | 513 | 100% |
|  | Y | 520 | 100% |
|  | T | 523 | 100% |
|  | Y | 533 | 100% |
|  | S | 536 | 100% |
|  | S | 609 | 100% |
|  | S | 610 | 100% |
|  | T | 681 | 100% |
|  | T | 738 | 100% |
|  | S | 750 | 100% |
|  | S | 777 | 100% |
|  | S | 810 | 100% |
|  | S | 821 | 100% |
|  | S | 824 | 100% |
|  | S | 831 | 100% |
|  | Y | 833 | 100% |
|  | T | 835 | 100% |
|  | T | 840 | 100% |
|  | S | 841 | 100% |
|  | T | 846 | 100% |
|  | T | 848 | 100% |
|  | Y | 853 | 100% |
|  | T | 854 | 100% |
|  | T | 861 | 100% |
|  | T | 868 | 100% |
|  | Y | 1146 | 100% |
|  | S | 1180 | 100% |
|  | Y | 1199 | 100% |
|  | S | 1230 | 100% |
|  | T | 1249 | 100% |
|  | T | 1251 | 100% |
|  | S | 1276 | 100% |
|  | S | 1278 | 100% |
|  | S | 1317 | 100% |
|  | S | 1343 | 100% |
|  | Y | 1354 | 100% |
|  | S | 1399 | 100% |
|  | S | 1400 | 100% |
|  | T | 1425 | 100% |
|  | S | 1433 | 100% |
|  | Y | 1524 | 100% |
|  | T | 1533 | 100% |
|  | S | 1535 | 100% |
|  | S | 1555 | 100% |
|  | S | 1565 | 100% |
|  | T | 1573 | 100% |
|  | S | 1588 | 100% |
|  | T | 1592 | 100% |
|  | Y | 1602 | 100% |
|  | T | 1609 | 100% |
|  | S | 1648 | 100% |
|  | S | 1655 | 100% |
|  | S | 1680 | 100% |
