# Supplementary material for: Petri Net-Based Model of Helicobacter pylori Mediated Disruption of Tight Junction Proteins in Stomach Lining during Gastric Carcinoma
Source: Front Microbiol. 2017 Sep 6;8:1682. doi: 10.3389/fmicb.2017.01682 (PMC5592237; doi:10.3389/fmicb.2017.01682)
Supplement: Supplementary file 3 [file Table2.docx]

Supplementary Material

Petri Net-based model of *Helicobacter pylori* mediated disruption of tight junction proteins in stomach lining during gastric carcinoma

Anam Naz^1^, Ayesha Obaid^1^, Faryal Mehwish Awan^1^, Aqsa Ikram^1^, Jamil Ahmad^2^, Amjad Ali^1*^

*** Correspondence:** Amjad Ali, amjaduni@gmail.com

# Supplementary Table 2

**Supplementary Table 2:** Rate parameters of transitions used within HPN models

| **Transition** | | **Rate of Transition** | **Transition** | **Rate of Transition** | **Description** |
| --- | --- | --- | --- | --- | --- |
| **Normal model** | | | **Disease model** | |  |
| t0 | 0.14 | | t16 | 0.4 | Describes the activation of CX32 in normal model through 3 different transitions (t0, t16 and t17) and its suppression (by t16 in disease) model. (14) |
| t1 | 0.5 | | t13 | 1 | Describes the over expression of PKC, MAPK and p38 in disease model. (53-56) |
| t2 | 1 | | t12 | 1 | Describes the activation of MAPK by t2 in normal model, whereas, 2 transitions (t12 and t13) results its over expression (54,56) |
| t3 | 0.5 | | t11 | 1 | t3 of normal model describes the activation of ERK protein during infection with half the rate of transitions (t11 and t12) which activate ERK in disease model (55,74) |
|  |  |  | t12 | 1 |  |
| t4 | 0.5 | | t10 | 1 | Describes the over expression of Raf protein under the influence of pathogenic proteins in disease model (55,74) |
| t5 | 0.5 | | t9 | 1 | Describes the over expression of Ras protein in disease model. (55,74) |
| t6 | 1 | | t8 | 1 | Describes the activation of CLDN2 (10,13) |
| t7 | 1 | | t5 | 1 | Describes the activation of IL1B and IL8 (50,52,70,71) |
| t8 | 1 | | t1 | 1 | Describes the activation of NF-kB normally by one transition (t8), whereas, overexpression is shown by 2 transitions (t1 and t4) in disease model (20,55,59,73) |
|  |  |  | t4 | 1 |  |
| t9 | 1 | | t3 | 1 | Describes the activation of PI3K |
| t10 | 1 | | t2 | 1 | Describes the activation of Akt |
| t11, t13 | 1 | |  |  | Describes the activation of TNFa |
| t12 | 1 | | t15 | 1 | t12 of normal model describes the activation of MLC and ZO1 by NF-kB, whereas in disease model t15 shows degradation of ZO1 (by inhibitory arc from cagA to ZO1) (56,60) |
|  |  |  | t7 | 1 | Describes the activation of MLC in disease model. (56) |
| t14 | 0.45 | | t18 | 0.08 | Describes the rate of degradation of CLDN2 which reduces in infection model than normal (10,72) |
| t15 | 0.1 | | t24 | 0.0001 | Describes the rate of degradation of CX32 which reduces in infection model than normal (14) |
| t16 | 0.41 | | t16 | 0.4 | t16 and t17 of the normal model shows the activation of CX32, whereas, t16 of disease model describes the suppression of CX32 during infection (11,14,47) |
| t17 | 0.15 | |  |  |  |
| t18 | 0.1 | | t17 | 1 | Describes the rate of degradation of ZO1 which increases in infection model than normal (2,4,59) |
