# Supplementary material for: Petri Net-Based Model of Helicobacter pylori Mediated Disruption of Tight Junction Proteins in Stomach Lining during Gastric Carcinoma
Source: Front Microbiol. 2017 Sep 6;8:1682. doi: 10.3389/fmicb.2017.01682 (PMC5592237; doi:10.3389/fmicb.2017.01682)
Supplement: Supplementary file 5 [file DataSheet2.DOCX]

Supplementary Material

Petri Net-based model of *Helicobacter pylori* mediated disruption of tight junction proteins in stomach lining during gastric carcinoma

Anam Naz^1^, Ayesha Obaid^1^, Faryal Mehwish Awan^1^, Aqsa Ikram^1^, Jamil Ahmad^2^, Amjad Ali^1*^

*** Correspondence:** Amjad Ali, amjaduni@gmail.com

# Supplementary Data

## Explanation of deductive reasoning used for modeling:

“Deductive reasoning is a basic form of valid reasoning. Deductive reasoning, or deduction start with a general statement, or hypothesis, and examines the possibilities to reach a specific logical conclusion. Deductive inference holds a theory on which predictions are made, if the theory is correct. Thus, it follows steps in such a way that, an inference is made based on premises. The form of deductive reasoning we followed in our study is based on logical modeling. Usually biological networks are quite complex and have many entities interacting with each other to produce a desired outcome. Therefore, we use a logical process in which a conclusion is based on the concordance of multiple premises that are generally assumed to be true. In our models, we assume only the change in expression of some important kinases in the presence of *H. pylori* virulence factors, which in turn can induce some specific PTMs in TJ proteins to change their behavior. To model these aspects, we abstracted the biological models to minimize the complexity that does not allow the computation of all solutions. Briefly, one such example is, if an entity A activates another entity B, which in turn activates C involved in interaction with another pathway, subsequently we can omit B and represent this relation as A activates C. In the process of reduction, the behavior of the removed entity B was preserved completely in the activation of C via A. This approach has been followed in our models to relieve the computational work load. One such example in our model the activation of NF-kB has been shown simply by the action of two kinases, however, there is a broad range of biological processes including innate and adaptive immunity, inflammation, stress responses, B-cell development, lymphoid organogenesis, action of proinflammatory cytokines, LPS, growth factors, and antigen receptors responsible of this single activation. All of these reactions cannot be shown in a single model so we deduce that in the presence of some specific kinases/protein, there will be a series of biological processes which finally activates NF-kB. Similar approach has been followed for other proteins/cytokines/kinase activation.”
